# Association Between Mini Nutritional Assessment and Health Related Quality of Life in Chinese Older Adults: A Large Cross-Sectional Study Stratified by Chronic Disease Status

**DOI:** 10.3390/nu17223510

**Published:** 2025-11-10

**Authors:** Gonghang Qiu, Zishuo Huang, Xuelan Zhao, Jiaoqi Ren, Borui Yang, Ziyi Wang, Hongfei Zhu, Shuna Lin, Liang Sun, Ying Wang, Houguang Zhou

**Affiliations:** 1 School of Public Health, Fudan University, Shanghai 200032, China; q18807039240@126.com (G.Q.); rowanh990207@gmail.com (Z.H.); 24211020249@m.fudan.edu.cn (B.Y.); wangzy201807@163.com (Z.W.); 23111020083@m.fudan.edu.cn (H.Z.); 23211020244@m.fudan.edu.cn (S.L.); 2 NHC Key Laboratory of Health Technology Assessment, Fudan University, Shanghai 200032, China; 3 Department of Geriatrics, Huashan Hospital, National Clinical Research Center for Aging and Medicine, Fudan University, Shanghai 200040, Chinarenjiaoqi1124@163.com (J.R.); 4 The NHC Key Laboratory of Geriatrics, Institute of Geriatric Medicine, Chinese Academy of Medical Sciences, Beijing Hospital/National Center of Gerontology of National Health Commission, Beijing 100730, China; sunbmu@foxmail.com

**Keywords:** Mini Nutritional Assessment (MNA), Health-Related Quality of Life (HRQoL), older adults, chronic diseases, comorbidity patterns, latent class analysis (LCA)

## Abstract

Background: Malnutrition among older adults in China demands greater attention due to its significant implications for both health and functional ability. However, the relationship between nutritional status and health-related quality of life (HRQoL), especially in the context of chronic diseases, remains underexplored in the Chinese elderly population. Methods: This large-scale cross-sectional study aimed to evaluate the association between nutritional status and HRQoL, stratified by chronic disease status, among 41,859 community-dwelling adults aged ≥ 65 years from 31 provinces in mainland China. Nutritional status was assessed using the Mini Nutritional Assessment (MNA), while HRQoL was measured using the SF-36 questionnaire; chronic disease status was based on physician diagnosis. Multiple linear regression was employed to examine the association between MNA and HRQoL, with subgroups defined by Latent Class Analysis (LCA) based on comorbidity patterns. Results: Results revealed significant positive associations between MNA scores and SF-36 total and domain scores among participants with chronic diseases (e.g., Total: β = 0.32, 95% CI: 0.04–0.60), but not among those without chronic diseases. LCA identified four comorbidity patterns: cardiovascular-rich, metabolic-rich, musculoskeletal-rich, and relatively healthy. Significant MNA-HRQoL associations were found in the cardiovascular (β = 0.58, *p* = 0.025), metabolic (β = 0.76, *p* = 0.022), and musculoskeletal (β = 1.01, *p* = 0.021) groups, but not in the relatively healthy group. Conclusions: These findings underscore the critical role of nutritional status in HRQoL among Chinese older adults with chronic diseases and highlight the need for tailored nutritional interventions in geriatric chronic disease management.

## 1. Introduction

The global population is aging at an accelerated pace, with projections indicating that the number of individuals aged 65 years and older will reach 2.2 billion by the year 2054 [1]. This rapid demographic shift has elevated geriatric health issues to a position of prominence within the global public health agenda. China currently holds the distinction of having the largest elderly population globally, a figure that continues to expand. From 2020 to 2050, China’s population aged ≥ 65 years old is estimated to more than double from 172 million to 366 million [2]. The rapid progression of population aging has been accompanied by a rising prevalence of unhealthy lifestyles among older adults, thereby exacerbating the complexity of geriatric health challenges. Consequently, there is an urgent need to prioritize and enhance the health management of this demographic group.

The World Health Organization (WHO) introduced the concept of “active ageing”, which emphasizes the optimization of opportunities for health, participation, and security in order to enhance HRQoL as individuals age [3]. The WHO defined HRQoL as an individuals’ perception of their position in life, situated within the context of their culture and value systems, and in relation to their goals, expectations, standards, and concerns [4]. Contemporary research consensus highlights that HRQoL among older adults is a multidimensional construct, encompassing physical, psychological, and social dimensions of health [5]. HRQoL is a multidimensional construct that serves as a comprehensive indicator of health status [6]. Nevertheless, when compared with developed countries, HRQoL scores in developing countries are generally lower [7]. This requires attention. Consequently, elucidating the factors associated with HRQoL and offering effective recommendations to enhance the HRQoL among the elderly hold substantial significance.

Nutrition is essential for sustaining life, supporting growth, and maintaining health through the ingestion and utilization of nutrients from food. This process involves not only obtaining energy and essential substances but also maintaining a dynamic balance of nutrients at molecular, cellular, and systemic levels, thereby regulating physiological functions and influencing disease risk. The relationship between nutritional status and HRQoL has garnered substantial attention, with numerous studies worldwide elucidating the intricate interaction between these two critical domains. For instance, a study comparing older adults residing in nursing homes with those receiving home-based care found that nutritional status was significantly correlated with HRQoL in both groups, alongside differences in physical activity levels [8]. A cohort study examining Singapore’s elderly population revealed that malnutrition is prevalent in approximately one-third of the older adults. In China, a multicenter hospital study across six provinces (Heilongjiang, Sichuan, Zhejiang, Hubei, Beijing, Qinghai) with 9966 participants and a rural cross-sectional survey with 1280 older adults both established significant nutrition-HRQoL associations [9,10]. Studies conducted in China, while indicative, remain limited to specific healthcare settings and regional populations, underscoring the need for broader epidemiological verification.

While the association between nutritional status and HRQoL has gained empirical support, the global epidemiological transition from infectious to non-communicable chronic diseases among aging populations has intensified disease burdens, which account for 74% of global mortality [11], particularly regarding multimorbidity. Emerging evidence indicates that compared with healthy older adults, patients with chronic disease exhibit poorer dietary quality, which significantly compromises their HRQoL and health outcomes. Furthermore, research demonstrates that patients with multimorbid score significantly lower across all SF-36 domains than non-comorbid counterparts, with clinically significant differences (twice the threshold) in physical functioning and mental health dimensions [12,13,14]. Based on these observations, we proposed the hypothesis that differences in chronic disease conditions may alter the relationship between nutritional status and HRQoL.

Therefore, utilizing a large, nationally representative database, this study aimed to investigate the nutritional status and HRQoL among older adults in China. The study population was stratified into healthy and chronic disease groups based on chronic disease status. Within the chronic disease group, latent class analysis (LCA) was further employed to identify distinct comorbidity patterns and examine differences across these subtypes. Through this approach, the study enhances the depth of research on malnutrition and HRQoL among older adults in China, offers tailored nutritional recommendations for those with specific multimorbidity profiles, and provides valuable insights for promoting holistic health and improving HRQoL in aging populations.

## 2. Material & Methods

### 2.1. Study Samples

China Ageing and Health Survey (CAHS) enrolled community-dwelling adults aged 65 years or older from mainland China (excluding Hong Kong, Macau, and Taiwan). To secure a nationally representative sample, a complex survey design was implemented, employing a stratified, multi-stage, probability-proportional-to-size (PPS) sampling strategy across 31 provincial-level administrative divisions. The sampling process unfolded across multiple stages: First, within each province, prefectural-level cities were stratified into high-, medium-, and low-economic tiers based on Gross Domestic Product (GDP) rankings. Subsequently, from each selected city, a mix of urban districts and rural counties was chosen to ensure urban-rural representation. Finally, communities were randomly selected within these counties/districts, resulting in the recruitment of 8 to 12 communities per province for the survey.

Eligible participants were community-dwelling older adults who had resided locally for at least six months. Individuals residing in institutional settings such as nursing homes, military bases, or hospitals were excluded. Deliberate efforts were made during recruitment to include vulnerable subgroups, including those living alone, with disabilities, or from empty-nest families, to mitigate selection bias and improve the generalizability of the sample at the community level. The initial dataset comprised 49,193 questionnaires. Through a multi-stage quality control process, which included the exclusion of incomplete or substandard responses and rigorous checks for logical errors and outliers, 7334 records were removed. Consequently, a final analytical sample of 41,859 participants was secured for the study. The geographical distribution of our survey sample across provinces is detailed in the Figure S1. In the final sample, there were no missing values for the independent or dependent variables. Among the covariates, marital status had 2915 missing values, personal monthly income had 5735 missing values, alcohol consumption had 4817 missing values, and smoking had 414 missing values. However, these missing values were addressed through multiple imputation methods, as detailed in the data analysis section.

### 2.2. Nutritional Status Assessment

The Mini Nutritional Assessment (MNA) was selected as it provides validated multidimensional evaluation of both overt malnutrition and preclinical risk factors, particularly suited for aging populations [15,16]. Trained investigators interviewed the participants. The MNA includes 18 questions grouped into 4 categories: anthropometry, general status, dietary habits and self-perceived health and nutrition states [17]. The total MNA score was calculated by summing the points from 18 items, with a possible range of 0 to 30, where a higher score indicates a better nutritional status [18,19,20].

### 2.3. Health Related Quality of Life Measurement

Health Related Quality of life (HRQoL) was quantified using the 36-Item Short Form Health Survey version 2 due to its robust psychometric properties in capturing both physical and mental health dimensions across cultures [21]. The instrument consists of 36 items grouped into the following eight domains: physical functioning (PF), role-physical (RP), bodily pain (BP), general health (GH), vitality (VT), social functioning (SF), role-emotional (RE), and mental health (MH). For each domain, the participant selected the response that best described their health status. The responses were then coded, summed, and transformed according to a standard algorithm to generate a score for each domain, ranging from 0 to 100, where a higher score indicates a more favorable health state [22]. With respect to the SF-36, the Physical Component Summary (PCS) and Mental Component Summary (MCS) scores were also calculated in addition to the scores of the eight scales. The SF-36 Total Score has been increasingly reported over the past two decades [23]. Therefore, we generated such a global measure by the arithmetic averaging of the scores of the eight domains.

### 2.4. Chronic Disease Assessment

The presence of chronic diseases was ascertained by trained investigators using a standardized protocol. Participants were presented with a pre-specified list of 154 chronic conditions and asked to report any that had been diagnosed by a physician or other healthcare professional. For each condition endorsed, the investigators confirmed that the participant was currently affected by or under treatment for the condition. This two-step process, facilitated by trained personnel, was designed to enhance reporting accuracy for both historical diagnosis and current disease status. The identification of comorbidity patterns included the following 13 diseases, which were selected for their high prevalence and significant impact on the health and functional status of the Chinese older adult population [24,25]: cerebral infarction, hypertension, coronary heart disease, heart failure, asthma, chronic obstructive pulmonary disease, malignant tumor of respiratory system, malignant tumor of digestive system, diabetes, hyperlipidemia, fracture, cervical spondylosis, lumbar disc herniation, osteoarthritis.

### 2.5. Covariate Assessment

Covariates were assessed through structured questionnaires, including gender (male/female), age (continuous), marital status (married/single), education (Primary school and lower, Junior high school, Senior high school or technical secondary school, College degree and above), monthly income (<3000¥, 3000–6000¥, 6000–10,000¥, ≥10,000¥), residence (urban/rural), smoking status (current smoker/non-smoker), alcohol consumption (current drinker/non-drinker), physical activity scale (continuous), bmi (continuous), depression (with/without depression).

### 2.6. Statistical Analysis

First, missing data in the covariates were handled prior to all analyses using Multiple Imputation by Chained Equations (MICE), generating 5 imputed datasets. All subsequent analyses were performed on these imputed datasets, and results were pooled according to Rubin’s rules.

Second, descriptive analysis was conducted to summarize the characteristics of the participants using frequencies and percentages for categorical variables and means with standard deviations for continuous variables. Subsequently, univariable linear regression analyses were performed to assess the crude associations between each sociodemographic characteristic and HRQoL, providing an overview of how HRQoL varies across different subgroups of the population.

Third, multiple linear regression models were utilized to examine the association between nutritional status, as assessed by the MNA, and HRQoL. The analysis adjusted for covariates, including gender, age, marital status, residence, education, monthly income, smoking status, and alcohol status. Then, stratified regression models were employed to evaluate the relationship between MNA and SF-36 results in different dimensions (Total score, MCS, PCS, PF, RP, BP, GH, VT, SF, RE, and MH). within subgroups defined by the presence or absence of chronic diseases. Results were visualized using forest plots, displaying effect estimates and 95% confidence intervals (CI). The analyses across SF-36 domains were conducted without formal correction for multiple comparisons; thus, the interpretation of domain-specific results would be further explored, and *p*-values would be interpreted with caution.

Finally, to further explore the association between nutritional status and HRQoL across different disease statuses, LCA was employed to identify the latent class structure within the study population and delineate the distinct patterns of comorbidity. The optimal number of latent classes was determined using four key indicators: Akaike Information Criterion (AIC), Bayesian Information Criterion (BIC), adjusted BIC (aBIC), and Entropy. Specifically, AIC, BIC, and aBIC collectively balance the model’s goodness of fit and structural complexity, with smaller values indicating superior model performance. The Entropy value (ranging from 0 to 1) is used to assess the clarity of class classification: a value closer to 1 reflects higher certainty in assigning individuals to their respective latent classes. Consistent with established methodological standards, a model was considered to have achieved a favorable classification effect when its Entropy value exceeded 0.8. To ensure the stability and reliability of the final LCA solution, we estimated the model using 500 random sets of starting values and 100 final stage optimizations. The best log-likelihood value was replicated successfully, indicating that the model converged on a global, rather than local, maximum and that the solution is stable.

We analyzed the association between nutritional status and HRQoL specifically within each of these comorbidity patterns. Statistical significance was set at *p* < 0.05. All statistical analyses were conducted using Stata 18.0 and Mplus 8.3.

## 3. Results

Basic characteristics of the 41,859 respondents are presented in Table 1. The mean score of HRQoL was 45.26. The largest demographics were female (*n* = 21,966, 52.48%), married (*n* = 33,152, 79.20%), living in urban areas (*n* = 25,553, 61.05%), with an education level of primary school or lower (*n* = 22,475, 53.69%), and having a monthly income below 3000¥ (*n* = 25,443, 60.78%). The majority did not smoke (*n* = 36,566, 87.36%) or drink alcohol (*n* = 38,840, 92.79%), and most had at least one chronic disease (*n* = 32,078, 76.63%). In terms of mental health, 62.29% (*n* = 26,075) had no depressive symptoms, while 29.64% showed mild depressive disorder.

Better HRQoL was associated with being male, being married, residing in urban areas, having higher education levels. Not smoking, drinking alcohol, having no or milder depressive symptoms, and higher BMI, PASE, and MNA scores were also linked to better HRQoL. All reported associations were statistically significant (*p* < 0.001) unless otherwise indicated in Table 1.

**Figure 1:**
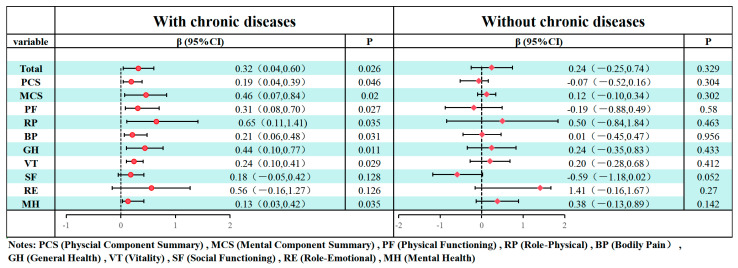
The fully adjusted model analyzing the relationship between MNA scores and HRQoL stratified by chronic disease status.

The results of the fully adjusted model analyzing the relationship between MNA scores and HRQoL stratified by chronic disease status are shown in Figure 1. Among participants with chronic diseases, MNA scores showed significant positive associations with most SF-36 domains and total scores (Total: β = 0.32, 95% CI: 0.04–0.60; PCS: β = 0.19, 0.04–0.39; MCS: β = 0.46, 0.07–0.84; PF: β = 0.31, 0.08–0.70; RP: β = 0.65, 0.11–1.41; BP: β = 0.21, 0.06–0.48; GH: β = 0.44, 0.10–0.77; VT: β = 0.24, 0.10–0.41; MH: β = 0.13, 0.03–0.42). In contrast, no significant associations were observed in participants without chronic diseases.

**Figure 2:**
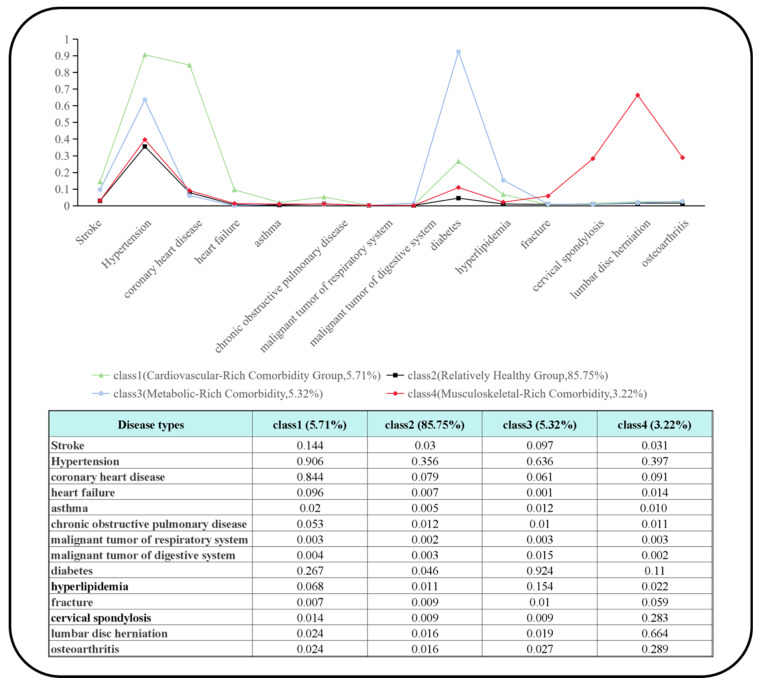
Graphical representation of the four latent classes by the probability of class membership of the chronic health conditions.

Table 2 presents the results of the LCA. Based on the model fit indices, four distinct comorbidity patterns were identified. Figure 2 illustrates the distribution probabilities of these four comorbidity patterns, which accounted for 5.71%, 85.75%, 5.32%, and 3.22% of the study population, respectively. These four comorbidity patterns were defined according to the maximum predicted probability of disease occurrence within each class, as follows: (a) Cardiovascular-Rich Comorbidity Group (class 1): Characterized by the highest prevalence of hypertension, coronary heart disease, heart failure, and other cardiovascular conditions; (b) Relatively Healthy Group (class 2): Exhibiting a lower prevalence of all assessed diseases compared to the other three groups; (c) Metabolic-Rich Comorbidity (class 3): Associated with a higher probability of diabetes and other metabolic disorders; (d) Musculoskeletal-Rich Comorbidity (class 4): Demonstrating a higher prevalence of osteoarthritis, fractures, lumbar disorders, spinal diseases, and other musculoskeletal conditions. Demographic and health-related characteristics across the four comorbidity patterns are summarized in Supplementary Table S1. Significant inter-class differences (*p* < 0.05) were found, underscoring their differences. Notably, the Cardiovascular-Rich group was relatively older (mean age: 75.64 years), the Metabolic-Rich group had a relatively higher mean BMI (24.53 kg/m^2^) and a greater proportion of urban residents (67.40%), while the Relatively Healthy group reported relatively higher physical activity levels (mean PASE: 101.63).

The associations between MNA and HRQoL across specific comorbidity patterns are detailed in Table 3. A significant positive association was observed between MNA scores and HRQol in three of the four identified comorbidity patterns. The most pronounced association was found in the Musculoskeletal-Rich Comorbidity group (class 4), where a higher MNA score was associated with better HRQoL (β = 1.01, 95%CI: 0.22–2.46, *p* = 0.021). Similarly, significant positive associations were also found in the Cardiovascular-Rich Comorbidity group (class 1) (β = 0.58, 95% CI: 0.16–1.01, *p* = 0.025) and the Metabolic-Rich Comorbidity group (class 3) (β = 0.76, 95% CI: 0.28–1.24, *p* = 0.022). In contrast, no significant association was found between nutritional status and HRQoL in the Relatively Healthy Group (class 2) (β = 0.18, 95% CI: −0.16–0.52, *p* = 0.270). These findings indicated a significant positive association between nutrition and HRQoL in individuals with specific multimorbidity profiles, particularly those involving metabolic, cardiovascular and musculoskeletal comorbidities, compared to their relatively healthy counterparts.

## 4. Discussion

This is the first study to investigate the association between nutritional status and HRQoL in China applying CAHS. Our analyses revealed a significant correlation between nutritional status and HRQoL. Notably, stratified by chronic disease status uncovered substantial heterogeneity in this association between individuals with and without chronic diseases. To further elucidate this heterogeneity, we performed LCA on the older adults with chronic diseases, ultimately identifying four distinct comorbidity patterns. Subsequent subgroup analyses demonstrated that the correlation between nutritional status and HRQoL also varied across these four comorbidity patterns. Collectively, this study holds important theoretical and practical significance, which could provide targeted dietary recommendations, offering guidance for improving nutrition and preventing chronic diseases among older adults. The following sections will discuss the aforementioned findings in detail.

Many studies conducted out of China have explored the relationship between nutritional status and HRQoL, and found that there was a positive correlation between nutritional status and HRQoL, which is consistent with our research results. This association has been documented in multiple countries, including Finland, Italy, Greece, and Turkey, using the MNA to evaluate nutritional status alongside validated HRQoL instruments such as the 15D, EQ-5D-3L, and SF-36 [26,27,28]. For example, a Finnish study of home-dwelling older adults found a moderate positive correlation between MNA scores and 15D-based HRQoL [27]. Similarly, in Greek older adults, better nutritional status was associated with significantly higher SF-36 scores, with well-nourished individuals exhibiting a 2.1-fold greater likelihood of improved HRQoL compared to those at risk of malnutrition or malnourished [28]. Collectively, these findings underscored a robust link between nutritional health and HRQoL across varied cultural and clinical settings. In China, several scholars have observed a positive correlation between nutritional status and HRQoL [9,10]. Previous research in China has often been limited by small sample sizes, raising questions about generalizability. In this context, our study of a representative older adults (mean age 74 years) with predominantly low socioeconomic status may further explore this association, underscoring its consistency in the Chinese population.

Stratified by chronic diseases status, we found that the association between nutritional status and HRQoL was not statistically significant in the absence of chronic diseases, whereas this association were more pronounced in the presence of chronic diseases. Older adults without chronic diseases exhibit a robust steady-state regulatory mechanism and flexible metabolic function. For them, the body compensates for minor daily nutrient imbalances through physiological mechanisms such as nutrient mobilization and metabolic adaptation, thereby preventing overt dysfunction [29,30]. Psychologically, their high resilience enables adaptation to these variations without compromising overall HRQoL [31]. Therefore, the lack of significant association between nutritional status and HRQoL is understandable.

Unexpectedly, among participants with chronic diseases, a 1-point increase in MNA score was significantly associated with a 0.32-point increase in the SF-36 Total score. The modest association gains public health importance when considering the potential for cumulative HRQoL improvement across China’s vast elderly population This observed association may be attributed to the following reasons: Firstly, older adults with chronic diseases are more susceptible to malnutrition. This heightened vulnerability in a multimorbid context is further underscored by studies identifying specific biochemical alterations, such as inflammation markers and nutritional markers, which are prevalent among malnourished geriatric inpatients with complex health profiles [32]. In such individuals, better nutritional status is consistently linked to better HRQoL, potentially by contributing to enhanced physical strength and ameliorated mood [33,34,35]. Secondly, individuals with chronic diseases often have a lower baseline HRQoL. In this context, better nutritional status is associated with better HRQoL, as it may be closely linked to the alleviation of symptoms and support of physical capacity [36,37]. Furthermore, we conducted a sub-dimensional analysis of the SF-36 scale, and the results for most dimensions were consistent with the total score results, indicating the robustness of our findings. Four comorbidity patterns were identified in the LCA. The finding indicated a significant positive correlation between nutritional status and HRQoL in the Cardiovascular-Rich Comorbidity Group, the Metabolic-Rich Comorbidity Group, and the Musculoskeletal-Rich Comorbidity Group, but no significant correlation was observed in the Relatively Healthy Group. The following content will expand upon this by discussing both the contributing factors and nutritional suggestions.

In the Cardiovascular-Rich Comorbidity Group, a 1-point increase in MNA score was significantly associated with a 0.58-point increase in SF-36 Total score. This association may be explained by pathways involving diet-induced inflammation, endothelial dysfunction, and oxidative stress, all of which are key pathways related to cardiovascular health [38,39,40]. Unhealthy dietary patterns, characterized by high intake of saturated fats and refined carbohydrates, can induce a chronic inflammatory state and endothelial dysfunction. This state is linked to impaired cardiac and vascular function and to symptoms such as chest pain and fatigue, which correspond with lower patients’ HRQoL. For patients with cardiovascular comorbidities, optimizing dietary structure is a key measure to improve HRQoL. Studies have confirmed the beneficial effects of the Mediterranean diet. This dietary pattern is featured by high consumption of vegetables, fruits, whole grains, legumes and nuts, moderate intake of fish and poultry, and restricted intake of red meat and processed foods. By providing abundant antioxidants such as polyphenols, dietary fiber and unsaturated fatty acids especially omega-3 fatty acids, the Mediterranean diet can effectively reduce systemic inflammatory response and regulate blood lipid and blood glucose levels [41].

In the Metabolic-Rich Comorbidity Group, a significant association was observed, with each 1-point MNA increase associated with a 0.76-point rise in HRQoL. This link may be driven by its impact on core aspects of metabolic disorders, such as insulin resistance, dyslipidemia, obesity, and hyperglycemia [42]. Unhealthy dietary patterns—particularly the long-term consumption of high-sugar, high-fat, and processed foods—can reduce insulin sensitivity, trigger abnormal expansion of adipose tissue, and induce chronic low-grade inflammation, which may further progress to metabolic syndrome and type 2 diabetes. These metabolic abnormalities directly contribute to symptoms such as fatigue, lack of energy, low mood, sleep disturbances, and reduced mobility, all of which severely impair patients’ daily functional capacity and HRQoL. For this population, increasing the intake of bioactive components such as polyphenols, dietary fiber, and polyunsaturated fatty acids, while limiting the consumption of saturated fatty acids, trans fats, refined carbohydrates, and added sugars, can significantly ameliorate the risk factors associated with metabolic syndrome [43]. This dietary optimization, in turn, contributes to improved HRQoL in affected patients.

A significant association was also found in the Musculoskeletal-Rich Comorbidity Group, where a 1-point MNA increase was associated with a 1.01-point gain in HRQoL. This association may involve its effects on skeletal muscle health, inflammatory response, and pain perception [44]. Malnutrition—especially the deficiency of key nutrients including protein, vitamin D, calcium, and magnesium—can exacerbate skeletal muscle protein catabolism and bone loss [45]. This further leads to reduced muscle strength, increased joint pain, and decreased bone mineral density, which in turn result in functional limitations, elevated fall risk, and a vicious cycle of chronic pain. Ultimately, these consequences collectively contribute to a further decline in HRQoL. For this population, the adoption of a high-protein dietary strategy is recommended, with an emphasis on consuming high-quality protein to preserve muscle mass and function [46]. Such high-quality protein sources include lean meat, fish, legumes, and dairy products. Additionally, adequate intake of calcium and vitamin D is essential for maintaining bone health; these nutrients can be obtained through dairy products, fortified foods, and sunlight exposure.

In summary, while existing research both domestically and internationally has established a consensus on the relationship between nutritional status and HRQoL, our study further explores population heterogeneity. We found that nutritional status and underlying mechanisms vary significantly between healthy individuals and those with different comorbidity patterns, leading to disparities in HRQoL. Consequently, tailored nutritional guidance should be developed based on specific comorbidity profiles.

## 5. Limitation

Our research has the following limitations. First, the cross-sectional nature of our study design precludes the determination of causal direction between nutritional status and HRQoL. Future longitudinal or interventional studies are required to establish causality. Second, the assessment of chronic diseases was based on self-reported physician diagnoses, which is susceptible to reporting bias. Older participants, particularly those with cognitive impairment, may misremember, underreport due to stigma or lack of access to healthcare, or confuse their conditions. Future studies could link survey data with electronic health records to obtain more objective diagnostic information and mitigate this potential bias. Third, the MNA is a validated screening tool for malnutrition risk but does not capture specific dietary patterns. This study can just offer dietary recommendations based on prior research experience but lacks empirical evidence. Thus, future research should incorporate detailed dietary assessments to elucidate the specific dietary components that drive the observed associations. Furthermore, additional variables such as ADL/IADL, oral health, or polypharmacy should be included to establish a more comprehensive understanding of these relationships. Finally, our analysis of multiple SF-36 outcomes was conducted without adjusting for multiple comparisons, which increases the risk of Type I error. Therefore, these findings should be interpreted as exploratory and hypothesis-generating. Future confirmatory studies should prioritize specific outcomes like the PCS and MCS with appropriate statistical adjustments.

## 6. Conclusions

This large-scale national cross-sectional study demonstrated a significant association between nutritional status and HRQoL among community-dwelling older adults in China. Notably, this association was moderated by chronic disease status: it remained robust among individuals with chronic diseases but was not observed in healthy participants. Among those with chronic diseases, latent class analysis identified four distinct comorbidity patterns. Specifically, nutritional status was significantly associated with HRQoL in the musculoskeletal comorbidity group, metabolic comorbidity group, and cardiovascular comorbidity group, but not in the relatively healthy subgroup. These findings underscore the necessity of integrating targeted nutritional screening and intervention into chronic disease management for older adults, with strategies tailored to their specific comorbidity profiles, to facilitate optimal healthy aging outcomes.

## Figures and Tables

**Table 1:** The HRQoL by individual level variables among Chinese older adults.

	N (%)/ Mean ± SD	Mean (SE)	95%CI for Mean	Regression β	*p*
Gender, n (%)					
Male	19,893 (47.52)	45.77 (0.068)	45.63, 45.90	ref	
Female	21,966 (52.48)	44.80 (0.066)	44.67, 44.93	1.16	<0.001 ***
Age, mean ± SD	74.13 ± 6.66	45.26 (0.047)	45.16, 45.35	−0.36	<0.001 ***
Marital status, n (%)					
Single	8707 (20.80)	42.61 (0.115)	42.38, 42.83	ref	
Married	33,152 (79.20)	45.95 (0.051)	45.85, 46.05	3.35	<0.001 ***
Residence, n (%)					
Urban	25,553 (61.05)	45.89 (0.060)	45.78, 46.01	ref	
Rural	16,306 (38.95)	44.26 (0.077)	44.11, 44.41	−1.63	<0.001 ***
Education, n (%)					
Primary school and lower	22,475 (53.69)	43.83 (0.067)	43.70, 43.95	ref	
Junior high school	10,402 (24.85)	46.98 (0.089)	46.80, 47.15	2.93	<0.001 ***
Senior high school or technical secondary school	6279 (15.00)	46.76 (0.117)	46.53, 46.99	3.15	<0.001 ***
College degree and above	2703 (6.46)	47.06 (0.176)	46.70, 47.39	3.22	<0.001 ***
Monthly income, Chinese Yuan, n (%)					
<3000	25,443 (60.78)	44.55 (0.062)	44.43, 44.67	ref	
3000~6000	12,959 (30.96)	46.43 (0.083)	46.26, 46.59	1.87	<0.001 ***
6000~10,000	2896 (6.92)	46.46 (0.171)	46.13, 46.80	1.91	<0.001 ***
≥10,000	561 (1.34)	44.04 (0.458)	43.14, 44.94	0.30	0.219
Smoking status, n (%)					
No	36,566 (87.36)	45.14 (0.051)	45.04, 45.24	ref	
Yes	5293 (12.64)	46.07 (0.132)	45.81, 46.33	0.93	<0.001 ***
Alcohol status, n (%)					
No	38,840 (92.79)	45.14 (0.049)	45.04, 45.24	ref	
Yes	3019 (7.21)	46.75 (0.176)	46.41, 47.09	1.61	<0.001 ***
Chronic disease Status, n (%)					
Without chronic disease	9781 (23.37)	44.20 (0.096)	44.01, 44.39	ref	
With chronic disease	32,078 (76.63)	45.27 (0.055)	45.17, 45.38	−0.002	0.799
Depression Status,					
No Depressive Symptoms	26,075 (62.29)	49.23 (0.045)	49.14, 49.32	ref	
Mild Major Depressive Symptoms	12,405 (29.64)	40.55 (0.079)	40.39, 40.71	−8.68	<0.001 ***
Moderate Depressive Symptoms	2524 (6.03)	33.40 (0.168)	33.07, 33.73	−15.83	<0.001 ***
Moderate-Severe Major Depressive Symptoms	638 (1.52)	28.84 (0.376)	28.10, 29.58	−20.38	<0.001 ***
Severe Major Depressive Symptoms	217 (0.52)	23.37 (0.796)	21.80, 24.94	−25.86	<0.001 ***
BMI	23.69 ± 3.46	-	-	0.16	<0.001 ***
PASE	100.58 ± 65.81	-	-	0.04	<0.001 ***
MNA	24.26 ± 3.17	-	-	0.54	<0.001 ***

Notes: ***: *p* < 0.001; BMI: Body Mass Index; PASE: Physical Activity Scale for the Elderly; MNA: Mini Nutritional Assessment; As BMI, PASE and MNA are continuous variables, they are not categorised. Consequently, their results are identical to those for Age, denoted by “-”, to prevent duplication.

**Table 2:** A comparison of the fit indices between one to six latent class models.

Number of Classes	AIC	BIC	ABIC	LMRT	BLRT	Entropy	Relative Frequence of Smallest Class (%)
1	190,552.220	190,673.209	190,628.717	-	-	-	-
2	187,887.798	188,138.418	188,046.256	0.0000	0.0000	0.630	13.91
3	186,830.499	187,210.750	187,070.918	0.0000	0.0000	0.728	2.27
4	186,155.710	186,665.591	186,478.089	0.0000	0.0000	0.833	3.22
5	185,941.510	186,581.022	186,345.850	0.0000	0.0000	0.587	2.12
6	185,863.059	186,632.203	186,349.360	0.0002	0.0000	0.593	1.06

Notes: AIC (Akaike Information Criterion), BIC (Bayesian Information Criterion), ABIC (adjusted BIC), LMRT (Lo-Mendell-Rubin Test), BLRT (Bootstrap Likelihood Ratio Test).

**Table 3:** Association of MNA with HRQoL in Specific Comorbidity Patterns.

Comorbidity Patterns	β (95% CI)	*p*-Value	Significance
Cardiovascular-Rich Comorbidity Group (class 1)	0.58 [0.16, 1.01]	0.025	*
Relatively Healthy Group (class 2)	0.18 [−0.16, 0.52]	0.270	-
Metabolic-Rich Comorbidity Group (class 3)	0.76 [0.28, 1.24]	0.022	*
Musculoskeletal-Rich Comorbidity Group (class 4)	1.01 [0.22, 1.74]	0.021	*

Notes: β: Standardized regression coefficient; *: *p* < 0.05; -: Not significant.

## Data Availability

The raw data supporting the conclusions of this article will be made available by the authors on request.

## Supplementary Materials

The following supporting information can be downloaded at: https://www.mdpi.com/article/10.3390/nu17223510/s1, Figure S1: The distribution of the number of provinces in the survey sample; Table S1: Comparison of demographic characteristics under different classes.
