# Supplementary material for: Association Between Mini Nutritional Assessment and Health Related Quality of Life in Chinese Older Adults: A Large Cross-Sectional Study Stratified by Chronic Disease Status
Source: Nutrients. 2025 Nov 10;17(22):3510. doi: 10.3390/nu17223510 (PMC12655801; doi:10.3390/nu17223510)
Supplement: Supplementary file 1 [file nutrients-17-03510-s001.zip › nutrients-3941043-supplementary.docx]

The geographical distribution of our survey sample across provinces is detailed in the Figure S1. The sample size varied considerably, reflecting the heterogeneous population distribution across the country. Notably, the most densely populated provinces, such as Shandong and Jiangsu, constituted the largest sample cohorts, each yielding over 2,063 participants and reaching up to 3,342. Conversely, regions with smaller populations, including Tibet Autonomous Region and Qinghai, contributed to the smallest sample group, ranging from 41 to 453 individuals. Intermediate sample sizes were observed in provinces such as Zhejiang (1,372-2,063 participants) and Shanxi (454-1,372 participants). This stratified distribution demonstrates that our survey successfully captured a wide geographic and demographic spectrum, thereby enhancing the representativeness and generalizability of our findings.


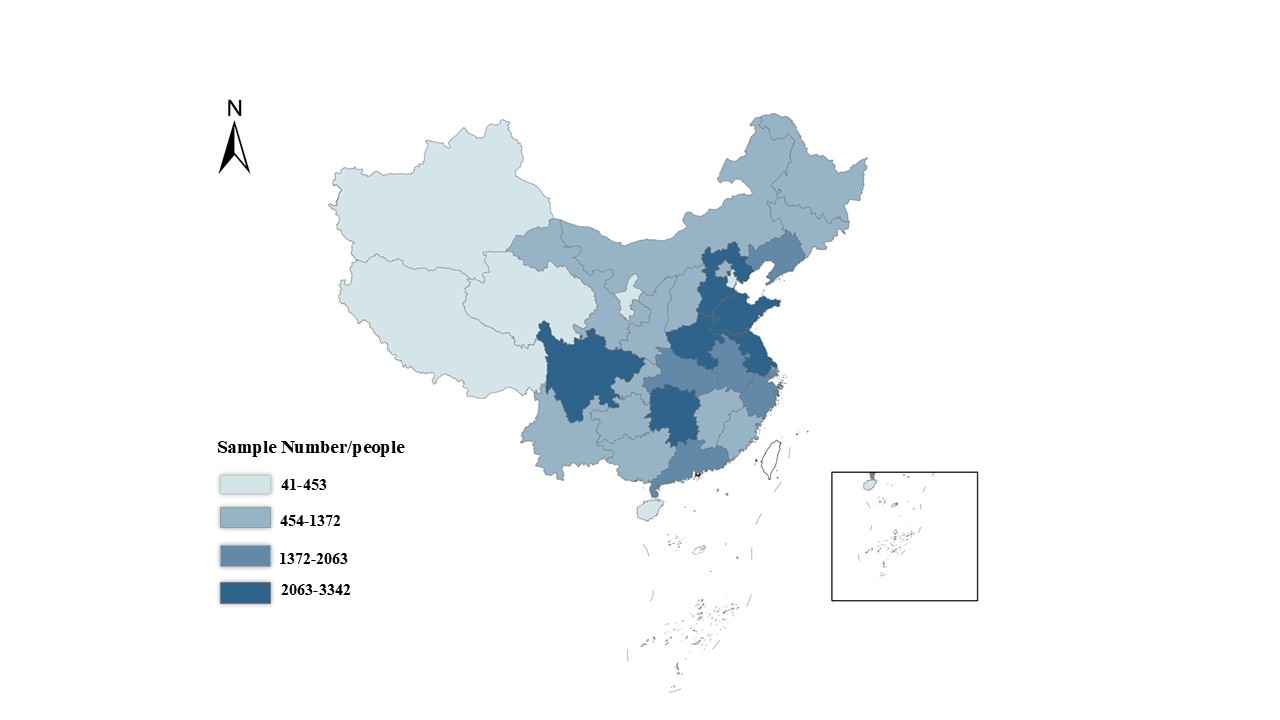


**Figure S1. The distribution of the number of provinces in the survey sample**

Table S1 presents the demographic and health-related characteristics across the four identified comorbidity patterns. The groups differed significantly in most characteristics (p < 0.05). The Cardiovascular-Rich group was relatively older, and a notably higher proportion of its members reported depressive symptoms. The Metabolic-Rich group had the highest mean BMI and the highest percentage of urban residents. In contrast, the Relatively Healthy group, which constituted the majority of the sample, exhibited the highest level of physical activity.

**Table S1 Comparison of demographic characteristics under different classes**

| **variable** | **total** | **class 1** | **class 2** | **class 3** | **class 4** | **P** |
| --- | --- | --- | --- | --- | --- | --- |
| **Sex,n (%)** |  |  |  |  |  | 0.177 |
| Male | 19893 (47.52) | 1091 (45.65) | 17106 (47.66) | 1040 (46.70) | 656 (48.66) |  |
| Female | 21966 (52.48) | 1299 (54.35) | 18788 (52.34) | 1187 (53.30) | 692 (51.34) |  |
| **Age,mean±SD** | 74.13±6.66 | 75.64±7.36 | 74.05±6.63 | 73.95±6.24 | 74.06±6.61 | <0.001 |
| **Marital status,n (%)** |  |  |  |  |  | <0.001 |
| Other | 8707 (20.80) | 607 (25.40) | 7350 (20.48) | 475 (21.33) | 275 (20.40) |  |
| Married | 33152 (79.20) | 1783 (74.60) | 28544 (79.52) | 1752 (78.67) | 1073 (79.60) |  |
| **Residence type,n (%)** |  |  |  |  |  | <0.001 |
| Urban | 25553 (61.05) | 1510 (64.18) | 21724 (60.52) | 1501 (67.40) | 818 (60.69) |  |
| Rural | 16306 (38.95) | 880 (36.82) | 14170 (39.48) | 726 (32.60) | 530 (39.31 |  |
| **Education level,n (%)** |  |  |  |  |  | <0.001 |
| Primary school and lower | 22475 (53.69) | 1249 (52.26) | 19400 (54.05) | 1102 (49.48) | 724 (53.71) |  |
| Junior high school | 10402 (24.85) | 594 (24.85) | 8876 (24.73) | 602 (27.03) | 330 (24.48) |  |
| Senior high school or  technical secondary school | 6279 (15.00) | 372 (15.56) | 5324 (14.83) | 374 (16.79) | 209 (15.50) |  |
| College degree and above | 2703 (6.46) | 175 (7.32) | 2294 (6.39) | 149 (6.69) | 85 (6.31) |  |
| **Monthly income,Chinese Yuan,n (%)** |  |  |  |  |  | <0.001 |
| <3000 | 25443 (60.78) | 1376 (57.57) | 22009 (61.31) | 1212 (54.42) | 846 (60.78) |  |
| 3000~6000 | 12959 (30.96) | 804 (33.64) | 10929 (30.45) | 829 (37.22) | 397 (29.45) |  |
| 6000~10000 | 2896 (6.92) | 163 (6.82) | 2480 (6.91) | 171 (7.68) | 82 (6.08) |  |
| ≥10000 | 561 (1.34) | 47 (1.97) | 476 (1.33) | 15 (0.67) | 23 (1.34) |  |
| **Smoking status,n (%)** |  |  |  |  |  | 0.033 |
| No | 36566 (87.36) | 2101 (87.91) | 31397 (87.47) | 1911 (85.81) | 1157 (85.83) |  |
| Yes | 5293 (12.64) | 289 (12.09) | 4497 (12.53) | 316 (14.19) | 191 (14.17) |  |
| **Alcohol status,n (%)** |  |  |  |  |  | 0.078 |
| No | 38840 (92.79) | 2212 (92.55) | 33347 (92.90) | 2039 (91.56) | 1242 (92.14) |  |
| Yes | 3019 (7.21) | 178 (7.45) | 2547 (7.10) | 188 (8.44) | 106 (7.86) |  |
| **Depression Status,n (%)** |  |  |  |  |  | 0.0499 |
| Without Depressive Symptoms | 26075 (62.29) | 1176 (49.21) | 22715 (63.28) | 1361 (61.11) | 823 (61.05) |  |
| With Depressive Symptoms | 15784 (37.71) | 1214 (50.79) | 13179 (36.72) | 866 (38.89) | 525 (38.95) |  |
| **BMI** | 23.69±3.46 | 23.98±3.26 | 23.62±3.48 | 24.53±3.23 | 23.69±3.64 | <0.001 |
| **PASE** | 100.58±65.81 | 91.69±65.52 | 101.63±65.90 | 94.02±64.18 | 99.37±64.71 | <0.001 |
| **Notes:**Cardiovascular-Rich Comorbidity Group (class 1);Relatively Healthy Group (class 2);Metabolic-Rich Comorbidity Group (class 3);Musculoskeletal-Rich Comorbidity Group (class 4) | | | | | | |
